# Intravenous Ascorbic Acid for the Prevention of Postreperfusion Syndrome in Orthotopic Liver Transplantation: Protocol for a Randomized Controlled Trial

**DOI:** 10.2196/50091

**Published:** 2023-12-15

**Authors:** Luis Gajate, Inés de la Hoz, Mercedes Espiño, Maria del Carmen Martin Gonzalez, Cristina Fernandez Martin, Ascensión Martín-Grande, Diego Parise Roux, Oscar Pastor, Judith Villahoz, Miguel Ángel Rodriguez-Gandía, Javier Nuño Vazquez

**Affiliations:** 1 Department of Anesthesiology and Critical Care Instituto Ramon y Cajal de Investigacion Sanitaria Hospital Universitario Ramon y Cajal Madrid Spain; 2 Department of Immunology Instituto Ramon y Cajal de Investigacion Sanitaria Hospital Universitario Ramon y Cajal Madrid Spain; 3 Department of Biochemistry Instituto Ramon y Cajal de Investigacion Sanitaria Hospital Universitario Ramon y Cajal Madrid Spain; 4 Department of Digestive Diseases Instituto Ramon y Cajal de Investigacion Sanitaria Hospital Universitario Ramon y Cajal Madrid Spain; 5 Department of Liver Surgery Instituto Ramon y Cajal de Investigacion Sanitaria Hospital Universitario Ramon y Cajal Madrid Spain

**Keywords:** antioxidant therapy, antioxidant, ascorbic acid, blood, controlled trials, hepatic, ischemia, ischemic, liver transplantation, liver, postreperfusion syndrome, randomization, randomized controlled trial, RCT, reperfusion injury, reperfusion, surgery, surgical, transplant, transplantation, vascular, vitamin C, vitamin, vitamins

## Abstract

**Background:**

Liver transplantation is the last therapeutic option for patients with end-stage liver disease. Postreperfusion syndrome (PRS), defined as a fall in mean arterial pressure of more than 30% within the first 5 minutes after reperfusion of at least 1 minute, can occur in liver transplantation as a deep hemodynamic instability with associated hyperfibrinolysis immediately after reperfusion of the new graft. Its incidence has remained unchanged since it was first described in 1987. PRS is related to ischemia-reperfusion (I/R) injury, whose pathophysiology involves the release of several mediators from both the donor and the recipient. The antioxidant effect of ascorbic acid has been studied in resuscitating patients with septic shock and burns. Even today, there are publications with conflicting results, and there is a need for further studies to confirm or rule out the usefulness of this drug in this group of patients. The addition of ascorbic acid to preservation solutions used in solid organ transplantation is under investigation to harness its antioxidant effect and mitigate I/R injury. Since PRS could be considered a manifestation of I/R injury, we believe that the possible beneficial effect of ascorbic acid on the occurrence of PRS should be investigated.

**Objective:**

The aim of this randomized controlled trial is to assess the benefits of ascorbic acid over saline in the development of PRS in adult liver transplantation.

**Methods:**

We plan to conduct a single-center randomized controlled trial at the Hospital Universitario Ramón y Cajal in Spain. A total of 70 participants aged 18 years or older undergoing liver transplantation will be randomized to receive either ascorbic acid or saline. The primary outcome will be the difference between groups in the incidence of PRS. The randomized controlled trial will be conducted under conditions of respect for fundamental human rights and ethical principles governing biomedical research involving human participants and in accordance with the international recommendations contained in the Declaration of Helsinki and its subsequent revisions.

**Results:**

The enrollment process began in 2020. A total of 35 patients have been recruited so far. Data cleaning and analysis are expected to occur in the first months of 2024. Results are expected around the middle of 2024.

**Conclusions:**

We believe that this study could be particularly relevant because it will be the first to analyze the clinical effect of ascorbic acid in liver transplantation. Moreover, we believe that this study fills an important gap in the knowledge of the potential benefits of ascorbic acid in the field of liver transplantation, particularly in relation to PRS.

**Trial Registration:**

European Union Drug Regulating Authorities Clinical Trials Database 2020-000123-39; https://tinyurl.com/2cfzddw8; ClinicalTrials.gov NCT05754242; https://tinyurl.com/346vw7sm

**International Registered Report Identifier (IRRID):**

DERR1-10.2196/50091

## Introduction

### Scientific Background and Explanation

The first liver transplant (LT) in the world was performed in 1963 [1], but the first successful transplant was carried out by Dr Starzl at the University of Pittsburgh in 1967 [2]. Until the introduction of immunosuppression with cyclosporine in the early 1980s, the survival rate of transplanted patients remained at 20% [3,4]. The 1-year and 5-year patient survival rates have increased to 85% and 70%, respectively, due to advances in surgical and anesthetic techniques and immunosuppression protocols. Despite these advances, hemodynamic changes during surgery remain a challenge for anesthesiologists.

One of the most serious changes during LT can occur at the time of graft reperfusion as an adverse hemodynamic situation known as postreperfusion syndrome (PRS), first described by Aggarwal et al [5] in 1987. They defined PRS as a deep and transient cardiovascular collapse immediately after graft reperfusion, with severe bradycardia, decreased mean arterial pressure (MAP) and systemic vascular resistance, severe arrhythmias, increased pulmonary artery pressure and central venous pressure, and even cardiac arrest. They defined it as a decrease in MAP of more than 30% within the first 5 minutes of graft reperfusion lasting at least 1 minute. The development of PRS is associated with decreased survival, an increased incidence of postoperative renal failure, early graft dysfunction, and the need for blood product transfusion in the setting of severe fibrinolysis [6-8]. Although it was first described in 1987, its incidence has not decreased over the past decades, ranging from 8% to 35% [9-11].

The intrinsic mechanisms of PRS are complex and not fully understood. Several theories have been proposed, including an abrupt accumulation of factors upon reperfusion, such as metabolic acidosis, hyperkalemia, hypokalemia, hypothermia, air emboli, and the release of vasoactive and proinflammatory mediators from both allograft and recipient, which are responsible for decreased systemic vascular resistance and myocardial depression [1,12-19]. During perfusion of the new graft, many proinflammatory cytokines are released from the new liver, such as tumor necrosis factor alpha and interleukins (ILs; IL-1, IL-2, and IL-8), and others from the patient (kallikrein and bradykinin). Other mediators have been found to be elevated after reperfusion (arginase, endotoxins, thromboxane, and monocyte chemoattractant protein-1), but the role of each of these mediators in the onset and severity of PRS is not known [18,20-24].

Ischemia-reperfusion (I/R) injury in LT is associated with graft dysfunction and decreased survival, but its exact role in PRS is controversial. I/R results in cell death due to lack of oxygenation, which occurs during the ischemic period and is exacerbated immediately after reperfusion by the release of proinflammatory cytokines and oxygen free radicals, activation of the complement system leading to sinusoidal vasoconstriction, and local hemoconcentration in the graft with the presence of neutrophils and platelets. All these mechanisms exacerbate the systemic inflammatory response (vasodilation and myocardial depression). The extent to which I/R contributes to the development of PRS and the severity of hemodynamic abnormalities is not entirely clear [25,26], but it seems logical to conclude that PRS is another manifestation of I/R injury. Several randomized controlled trials have been published using different drugs to prevent the onset of PRS (methylene blue, acetylcysteine, antifibrinolytics, and others). However, none of these have shown a beneficial effect.

On the other hand, there has recently been renewed interest in the use of high-dose vitamin C as a supportive treatment in patients with severe septic shock (metabolic resuscitation) [27]. Sepsis has been shown to decrease the levels of some vitamins, such as ascorbic acid, which has been associated with the development of multiorgan failure and death. Intestinal absorption of vitamin C is saturable, so the only way to increase its levels would be by intravenous administration. Tanaka et al [28] reduced resuscitation fluid volumes in patients with burns by administering high doses of intravenous ascorbic acid. This was a randomized controlled trial of 37 patients allocated to receive a dose of 66 mg/kg/hour of vitamin C versus saline during the first 24 hours of resuscitation. Although MAP and central venous pressure were similar at the end of the vitamin C or saline infusion, fluid administration was lower in the intervention group, and patients maintained higher urine output and developed less wound edema with a significant reduction in the duration of mechanical ventilation (12 days vs 21 days; *P*=.03).

Zabet et al [29] investigated the effect of high doses of vitamin C on vasopressor requirements in septic shock. As ascorbic acid is required for catecholamine synthesis [30], it was investigated whether it could be useful in shortening vasopressor weaning. A total of 24 patients with vasopressor-dependent septic shock were randomized to receive vitamin C at a dose of 25 mg/kg intravenously every 6 hours versus saline. The dose and duration of vasopressors were significantly reduced in the intervention group, as was mortality.

The most important study in septic patients was conducted by Marik et al [31], who used a combination of hydrocortisone, ascorbic acid (1.5 g every 6 hours), and thiamine (200 mg every 12 hours) in 47 patients with sepsis admitted to the intensive care unit with procalcitonin levels >2 ng/mL. The aim of thiamine was to prevent kidney oxalate accumulation. Mortality decreased in patients treated with vitamin C (*P*<.001). They were weaned from vasopressors earlier and received less intravenous fluid for resuscitation than the control group.

Vitamin C is safe even in very high doses. Fowler et al [32] conducted a safety study of intravenous ascorbic acid in 24 patients with severe sepsis (low dose 12.5 mg/kg every 6 hours vs high dose 50 mg/kg intravenously every 6 hours). No adverse events were observed. No adverse effects were reported in any of the above studies, including the study by Tanaka et al [28], in which the doses were very high (1.6 g/kg over 24 hours). Metabolism of vitamin C to oxalic acid could theoretically cause calcium oxalate nephropathy. However, this does not seem to be a real problem, as renal toxicity is dose-dependent and has only been reported at very high doses (>40 g/day) [33]. In addition, coadministration of thiamine would reduce the conversion of vitamin C to oxalate. In the study by Marik et al [31], the incidence of renal failure requiring renal replacement therapy was significantly lower in the intervention group (33% vs 10%; *P*=.02).

However, several meta-analyses have been published in recent years with mixed results. In most of them, a reduction in mortality, duration of mechanical ventilation, and hospitalization could not be demonstrated. However, a reduction in the sepsis-related organ failure assessment score and a shorter duration of treatment with vasoactive drugs is a common finding [34-38].

I/R injury plays a pivotal role in the development of graft dysfunction after liver transplantation, and excessive free radical production and massive depletion of endogenous antioxidants are common mechanisms underlying I/R injury. Ascorbic acid is an essential micronutrient involved in the antioxidant response and modulation of apoptosis and inflammation. These properties may, therefore, make it a potential strategy for reducing I/R injuries. In addition, its safety profile, low cost, and ease of administration favor its use in this scenario [39]. As discussed above, we can consider PRS as one of the clinical manifestations of I/R injury. Therefore, if we can prevent or at least attenuate I/R injury, we may be able to reduce the incidence of I/R injury.

### Aim

The aim of the study is to determine the possible benefit of vitamin C in reducing the incidence of PRS in liver transplantation.

## Methods

### Study Setting

**Figure 1:**
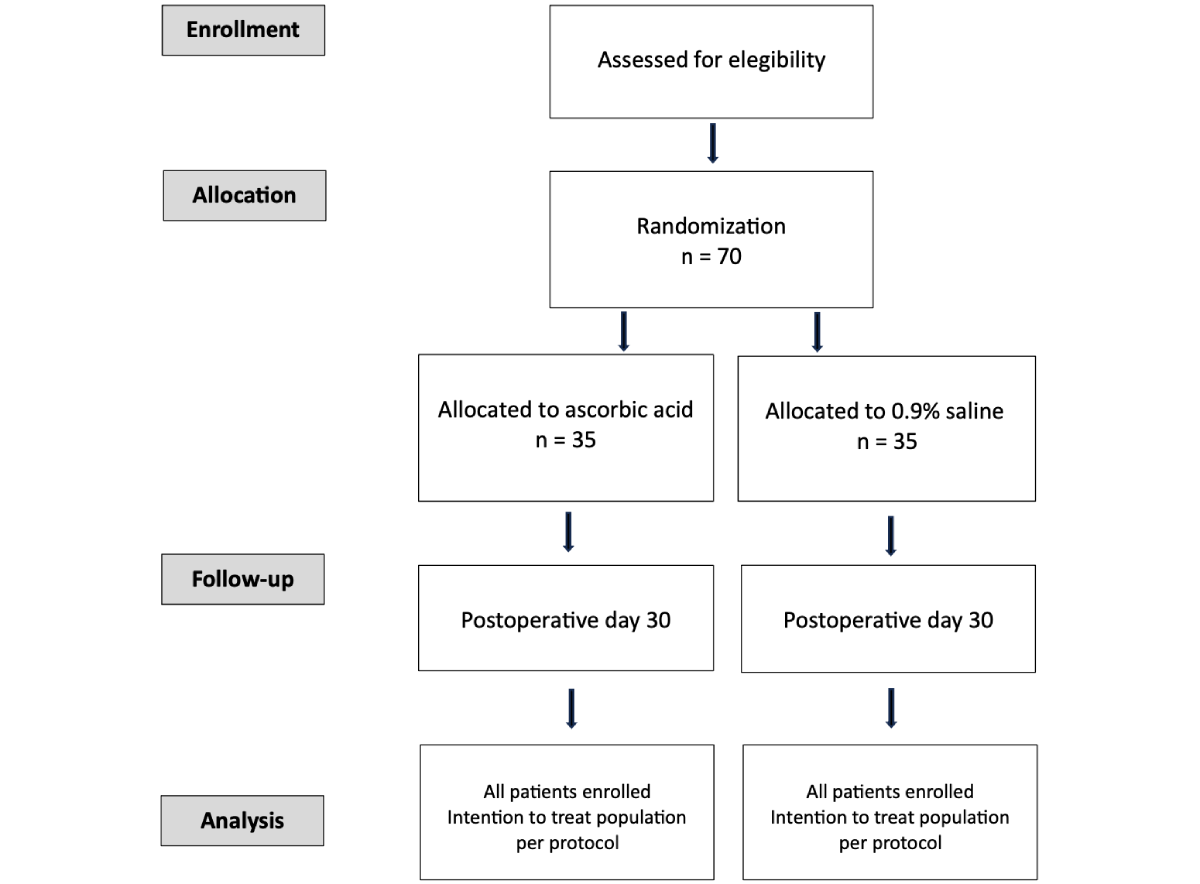
CONSORT (Consolidated Standards of Reporting Trials) flow diagram.

The trial has been designed in accordance with the updated CONSORT (Consolidated Standards of Reporting Trials), the Declaration of Helsinki, and the Good Clinical Practice guidelines issued by the Spanish regulatory authorities. The study will be conducted at a single center, the Hospital Universitario Ramon y Cajal in Madrid (Figure 1).

### Trial Design

This study is designed as a single-center, double-blind, randomized controlled trial. Participants will be randomized to receive ascorbic acid (intervention group) or saline (0.9%; control group). We will conduct a superiority analysis.

### Eligibility Criteria for Participants

All patients over 18 years of age who are on the waiting list for liver transplantation may be eligible to participate in this study. The inclusion and exclusion criteria are detailed in Textbox 1.

**Inclusion and exclusion criteria.**

**Inclusion criteria**

- Aged between 18 and 67 years
- Waiting for a liver transplant
- Negative pregnancy test in women

**Exclusion criteria**

- Pregnancy
- Allergy to ascorbic acid
- Nephrolithiasis
- Glucose-6-phosphate dehydrogenase (G6PD) deficiency
- Hyperoxaluria
- Hyperuricemia
- Haemochromatosis
- Sickle cell anemia
- Serum creatinine >1.2 mg/dL (women) and 1.3 mg/dL (men)
- Split liver graft
- Acute liver failure
- Living donor liver transplantation
- Grafts from donation after circulatory death
- Treatment with indinavir, vitamin B_12_, cyclosporine, iron, deferoxamine, or disulfiram

### Study Procedures and Intervention

The intervention group will receive a dose of 1500 mg of intravenous ascorbic acid (vitamin C) diluted in 100 mL of 0.9% saline for 30 minutes, to be administered during the anhepatic phase of liver transplantation.

The control group will receive a dose of 100 mL of 0.9% saline for the same period and under the same conditions.

### Outcome Measurements

The primary end point of the study is the percentage of patients who develop PRS after liver graft reperfusion. PRS is defined as a 30% decrease in MAP within the first 5 minutes after portal-clamp release that persists for at least 1 minute.

Textbox 2 contains the secondary variables that are also analyzed.

**Secondary variables.**

**Secondary variables**

- The percentage of patients with primary failure and early graft dysfunction. Primary graft failure is defined as the complete absence of liver graft function. The patient will not survive without retransplantation. In the case of early graft dysfunction, patient survival is likely and possible without retransplantation, although morbidity will be greater than in the case of normal function. The following Olthoff criteria are used for its definition (one criterion is enough):
  Bilirubin ≥10 mg/dL on postoperative day 7.
  International normalized ratio ≥1.6 on postoperative day 7.
  Alanine aminotransferase or aspartate aminotransferase >2000 IU/mL in the first 7 postoperative days.
- Length of stay in the intensive care unit and hospitalization. The number of days spent in the surgical intensive care unit and the total number of days spent in the hospital.
- Dose and duration of catecholamines from the time of reperfusion.
- Length of time on mechanical ventilation (hours). Time to extubation of the patient.
- Vitamin C levels before transplantation and 12 hours after transplantation. Measurements are performed by high-pressure liquid chromatography with an ultraviolet detector.
- Levels of inflammatory markers (IL-1β, tumor necrosis factor alpha, IL-6, IL-8, and IFN-γ) before and after transplantation as measured by the high-sensitivity enzyme-linked immunosorbent assay technique.
- Development of postoperative kidney failure (acute kidney injury [AKI]) during the first week. AKI is defined as an increase in serum creatinine (SCr) ≥0.3 mg/dL within 48 hours or an increase in SCr × 1.5 times baseline; known or presumed to have occurred within the previous 7 days.
- Need for postoperative renal replacement therapy during the first week. Need for any type of renal replacement therapy during the first 7 days after transplantation, regardless of the technique used.
- Graft and patient survival 30 days after transplantation.

### Participant Timeline

Patients will be monitored from randomization until day 30 post surgery. The team will collect variables daily through postoperative day 7 and a final visit on postoperative day 30. The research team will closely monitor adverse events, including onset, duration, intensity, progression, and outcome, to assess their relationship to the drug.

### Sample Size Calculation

The current incidence of PRS at our center is 30%. Assuming a 50% decrease in PRS incidence (from 30% to 15%) with an α risk of 5% and a power of 80%, bilateral testing, and the Fleiss correction with a 10% loss rate, we require 268 participants (134 in each group). Due to the limited number of transplants per year in our center, achieving such a large sample will take 6-8 years. Given that this study will be conducted at a single center, the projected patient enrollment is 35 individuals per year, resulting in a final sample size of 70 participants. To attain 80% power with this sample, it is necessary to achieve a complete elimination of PRS. Although it is unlikely that such a reduction in the incidence of PRS would be achieved, we believe that this is a reasonable number of participants to show differences in the primary outcome that, although not reaching the desired statistical significance, would serve to justify a future multicenter randomized controlled trial. Participants will be randomly assigned to groups using a computer-generated sequence created by SPSS Statistics (SPSS Inc). The computer-generated sequence will allocate 35 participants to the ascorbic acid intervention group and 35 participants to the saline control group. To ensure independence, an unrelated staff member will enroll patients and prepare medication or saline upon arrival.

### Analysis

Quantitative variables will be described using mean, median, SD, IQR, 95% CI, and range values. Quantitative data will be presented as absolute and relative frequencies. The significance level in any statistical test will be set at α=.05. Both groups will be assessed to ensure they are balanced in all variables.

A total of 2 populations will be established: one based on intention to treat and the other based on efficacy. The first population will consist of all participants randomized at the start and divided into 2 groups according to the corresponding randomization. The second population will consist of the participants who complete the protocol and end up in the same group in which they were randomized. All analyses will be conducted on both populations; however, the primary analysis will focus on the intention to treat the population. Statistical analyses will be performed using SPSS Statistics.

The comparison between participants who experienced PRS during the intervention will be performed using the chi-square test. Cohort proportions are compared using relative risk with 95% CIs. In a secondary analysis, we will adjust for potentially confounding factors related to the main outcome variable by using a multivariate technique. These factors include variables that showed significant differences (with *P*≤.20) between the control and treatment groups and have been identified as potential confounds. When dealing with binary factors, we will assess the magnitude of association with odds ratios, while for ordinal or quantitative factors, we will use the Spearman correlation coefficient. A binary logistic regression analysis will be conducted to examine the presence of PRS. The control variable (ascorbic acid vs saline) and all variables resulting from the previous univariate analysis will serve as independent variables. The same procedure will be applied to secondary efficacy and safety analyses.

### Monitoring and Quality Assurance

The data collected in the study will be coded, and only the principal investigator and collaborators will be able to access this information and link it to each patient’s clinical history. The information will be stored in a database that adheres to current regulations on personal data protection, including Regulation (EU) 2016/679 of the European Parliament and of the Council of April 27, 2016, on Data Protection, and the Organic Law 3/2018 of December 5, on Personal Data Protection and Guarantee of Digital Rights. Only the data included in the medical file and related to the study will be subject to verification. This verification will be carried out in the presence of the principal investigator or collaborators, who have the responsibility to protect the privacy of data within the clinical records of the participants who take part in the clinical trial.

Therefore, the identity of the participant will not be disclosed to any other person except to the health authorities, when required, or in cases of medical emergencies. The Research Ethics Committees, the representatives of the Health Authority in matters of inspection, and the personnel authorized by the sponsor will only have access to verify the personal data, the procedures of the clinical trial, and compliance with good clinical practice (always maintaining the confidentiality of the information). The principal investigator and the sponsor will keep the data collected for the study for at least 25 years after its completion. Under no circumstances will the identities of study participants be disclosed at the time of publication of the results.

### Ethical Considerations

This study has been registered in the European Union Drug Regulating Authorities Clinical Trials Database (EudraCT; 2020-000123-39) and at ClinicalTrials.gov (NCT05754242). The Ethics Committee of Hospital Universitario Ramón y Cajal and the Spanish Medicines and Health Products Agency (Agencia Española del Medicamento y Productos Sanitarios [AEMPS]) have both approved this study protocol (AEMPS-20-0052).

This clinical trial will adhere to the ethical principles governing biomedical research on human beings and respect their fundamental rights in accordance with international recommendations included in the Declaration of Helsinki and its latest revision of Fortaleza (Brazil, 2013). The guidelines of AEMPS will also be followed, in addition to following national recommendations.

Participants will be given both oral and written information about how the study will be conducted, its objectives, the potential risks involved, and the guidelines to be followed during the monitoring period. If a patient satisfies the inclusion criteria and agrees to participate in the study, they will provide written informed consent. They are free to withdraw their consent and discontinue the study at any point and for any reason.

## Results

The enrollment process began in 2020. A total of 35 patients have been recruited so far. Data cleaning and analysis are expected to occur in the first months of 2024. Results are expected by mid-2024.

## Discussion

### Overview

This study aims to show if ascorbic acid could be useful in preventing PRS during liver transplantation. Improved hemodynamic stability following graft reperfusion, combined with the potential antioxidant effects of vitamin C, may lead to enhanced outcomes in liver transplantation.

The potential protective effect of adding ascorbic acid to transplant preservation solutions before transplantation has been studied with promising results. However, there has been limited research on the benefits of intravenous vitamin C administration for the prevention of I/R injury in solid organ recipients. PRS may be considered a severe clinical manifestation of I/R injury in liver transplant recipients [25,26]. Therefore, we propose that ascorbic acid’s antioxidant properties may play a protective role in preventing PRS during liver transplantation.

Vitamin C acts as an antioxidant. It reduces inflammation and modulates the immune response. It also increases the synthesis and sensitivity of endogenous vasopressors, acts as an enzymatic cofactor, and has bacteriostatic and antithrombotic properties [29].

The hallmark of PRS is a depressed state of the cardiovascular system. The pathophysiological mechanisms underlying PRS are thought to involve the release of proinflammatory mediators with vasodilatory effects by both graft and recipient [20-24]. Therefore, vitamin C may have beneficial effects in the prevention and treatment of I/R injury, potentially reducing the incidence of PRS.

We are aware that studies following Marik et al’s [31] publication have reduced enthusiasm for the use of vitamin C in the treatment of patients with severe sepsis. In fact, its use is no longer recommended by the Surviving Sepsis Campaign guidelines. However, recent publications, including several meta-analyses, confirm the beneficial effects of this treatment in septic patients. These effects include reducing the number of days on vasopressors and improving the sepsis-related organ failure assessment score [34-38]. We believe that the administration of ascorbic acid to patients with sepsis could potentially have a positive impact, especially considering its minimal side effects. Additional prospective clinical trials are needed to definitively establish the benefits of this treatment in this specific patient population.

Likewise, treating and preventing I/R injury in solid organ transplant recipients may be helpful, given the beneficial effects of ascorbic acid demonstrated in some of these studies in patients with sepsis.

This study is of particular importance because it is the first to evaluate the effects of administering ascorbic acid before reperfusion of the new liver graft to prevent PRS. It will also provide more information about the levels of inflammatory markers before and after reperfusion and how they change with the onset of PRS.

If the study shows positive results, the ideal dose to be used and the exact duration of treatment should be defined and could be applied to other types of solid organ transplantation.

This study aims to address the current gap in knowledge regarding the clinical effectiveness of administering vitamin C during solid organ transplantation, particularly in liver transplantation. We will provide valuable insights into this area of research through rigorous data analysis.

### Strength and Limitation of the Study

The main limitation of the study, due to the limited number of participants planned, is that it may not achieve adequate statistical power to establish with sufficient scientific evidence whether vitamin C reduces the PRS and improves the results of LT. However, we believe that if positive results in favor of vitamin C are obtained, the trial would serve as the basis for a larger trial involving other transplant centers, which would allow us to demonstrate the benefit of vitamin C with enough statistical power. It is possible that we may not be able to control for additional variables that may bias the results, including those related to the surgical and anesthesia teams. A forthcoming multicenter trial could address this issue.

## Abbreviations

AEMPS: Agencia Española del Medicamento y Productos Sanitarios
CONSORT: Consolidated Standards of Reporting Trials
I/R: ischemia-reperfusion
IL: interleukin
LT: liver transplant
MAP: mean arterial pressure
PRS: postreperfusion syndrome

## Data Availability

The data sets used or examined in this study will be accessible from the corresponding author upon request.
